# One-Dimensional and Two-Dimensional Zn(II) Coordination Polymers with Ditopic Imidazo[1,5-*a*]pyridine: A Structural and Computational Study

**DOI:** 10.3390/molecules29030653

**Published:** 2024-01-30

**Authors:** Mattia Sozzi, Michele R. Chierotti, Roberto Gobetto, Rosa M. Gomila, Vittoria Marzaroli, Emanuele Priola, Giorgio Volpi, Stefano Zago, Antonio Frontera, Claudio Garino

**Affiliations:** 1 Department of Chemistry and NIS Centre, University of Turin, Via Pietro Giuria 7, 10125 Torino, Italy; 2 Department of Chemistry, Universitat de les Illes Balears, Crta de Valldemossa km 7.5, 07122 Palma de Mallorca, Spain

**Keywords:** coordination polymers, imidazo[1,5-*a*]pyridine, imidazopyridine, zinc, dicarboxylic acid

## Abstract

Zn(II) coordination polymers are being increasingly studied for their stability and properties. Similarly, there is a growing interest in imidazo[1,5-*a*]pyridine derivatives, which show great potential in luminescence and pharmaceutical applications. In this work, we successfully synthesized and crystallized three new coordination polymers, using Zn(II) as the metallic node, dicarboxylic acids of different length and nature as linkers, and a linear ditopic imidazo[1,5-*a*]pyridine derivative, to explore the role of this molecule as a propagator of the dimensionality of the structure or as an ancillary ligand. Our work demonstrates the structural capability of imidazo[1,5-*a*]pyridines in an unexplored domain for this family of ligands. Notably, we observed a pronounced ability of this heterocyclic scaffold to establish π···π interactions in the solid state. The supramolecular π-stacked assemblies were theoretically analyzed using DFT calculations based on model structures.

## 1. Introduction

Coordination polymers (CPs), formed through the combination of metal ions or clusters with organic ligands, constitute a diverse class of promising materials, extensively applied in many fields such as gas storage and separation, catalysis, magnetism, and drug delivery [1,2,3,4,5,6,7]. Moreover, CPs represent a class of crystalline materials resulting from the combination of metal ions or clusters with a wide array of organic ligands via coordination bonds. These materials exhibit a wide range of properties and applications due to tunable structures, high porosity, and diverse chemical compositions. In this context, different kinds of organic linkers have been investigated. Examples include carboxylate ligands (e.g., terephthalic, fumaric, benzenetricarboxylic acids, etc.) and porphyrin-based macrocycles (known for their ability to coordinate with metal ions in a planar fashion); bipyridines and terpyridines (containing multiple pyridine units linked with different orientations); pyrazolate ligands (well-known nitrogen donors with versatile coordination chemistry that can act as neutral/anionic monodentate or as exo/endo-bidentate anionic ligands); and many other linkers employed to impart specific properties and variable geometries to CPs.

When it comes to the selection of metal ions for the construction of CPs, zinc can be considered as an affordable, abundant, non-toxic, and environmentally friendly element. In the context of this work, the Zn(II) ion is particularly well-suited for the formation of coordination polymers and networks. Indeed, the spherical d^10^ electronic configuration of this cation is associated with a flexible coordination environment, compatible with several different geometries. Depending on the selected linkers, the metal node can vary the coordination geometry from tetrahedral to trigonal bipyramidal, to octahedral and corresponding distortions from the ideal polyhedron, thus allowing for a large variety of architectures, resulting from the self-assembly of the Zn(II) ion with different organic ligands [8]. Thanks to its versatility, zinc is an important element in different functional materials. For instance, zinc-based materials represent a class of promising and cost-effective electrocatalysts for CO_2_ reduction [9] or photoelectrochemical catalysts [10]. Ongoing efforts are currently dedicated to the development of zinc-based materials for applications in energy storage [11], optoelectronics, and nonlinear optics [12,13,14,15,16]. Furthermore, the well-known biocompatibility and antimicrobial properties of zinc pave the way for innovative biocompatible materials for medical and pharmaceutical applications [17,18]. In the specific case of this study, we propose a few CPs based on Zn(II) as the metallic node, dicarboxylic acids of different length and nature as linkers, and a linear ditopic chelating ligand (L), based on the imidazo[1,5-*a*]pyridine core, serving a dual role of either propagator of the dimensionality of the structure or ancillary ligand. To the best of our knowledge, this work reports the first example of Zn(II)-coordination polymers incorporating an imidazo[1,5-*a*]pyridine derivative within their framework. The imidazo[1,5-*a*]pyridine represents a peculiar N-based heterocyclic skeleton in which two aromatic rings (namely imidazole and pyridine) are fused together with a specific orientation. The resultant aromatic core has unique chemical properties and has been extensively employed in various technological fields including lightening technologies, solar energy conversion, and storage systems, as well as pharmaceuticals and biologically active molecules [19,20,21].

**Scheme 1:**
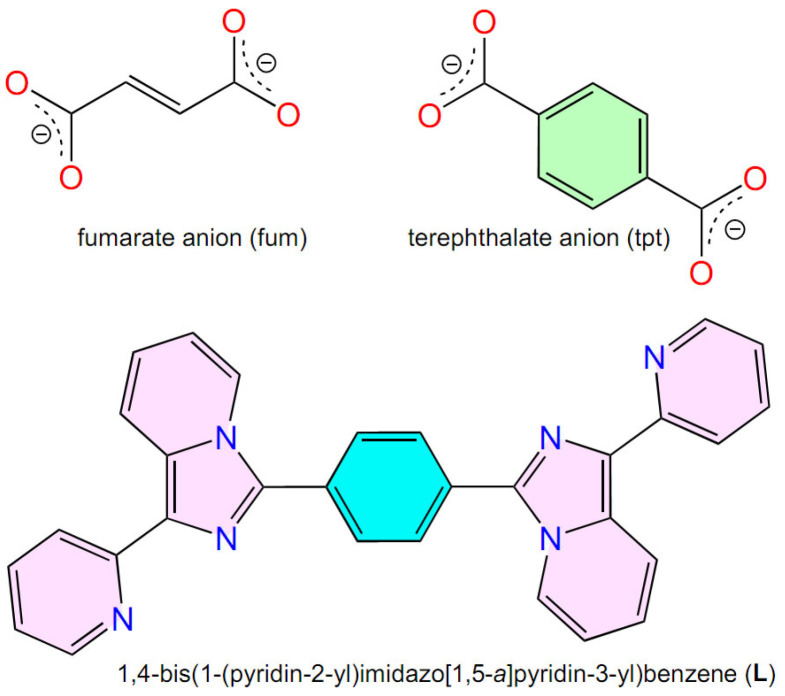
Chemical structures of the fumarate and terephthalate linkers and the ditopic pyridinyl imidazo[1,5-*a*]pyridine ligand (in pink, the imidazo[1,5-*a*]pyridine core and the pendant pyridinyl substituent).

When conveniently substituted, imidazo[1,5-*a*]pyridine derivatives can easily act as chelating ligands for various metal ions, including but not limited to Zn(II), Cu(I and II), Ir(III), Ni(II), and Co(II), and numerous other cations, as widely reported in the literature [22,23,24,25,26,27,28]. Specifically, the imidazo[1,5-*a*]pyridine core, when substituted in 1 with a pendant pyridine, guarantees the typical N–N bidentate ligand motif, which is well-known for allowing suitable complexation reactions with different metals [29,30,31]. Alternatively, in a different scenario, this heterocyclic skeleton can function as a chelating N–O ligand if an appropriate substituent is opportunely introduced [22,23,32]. As a matter of fact, this interesting class of heterocyclic aromatic compounds could be as versatile as the overused families of bipyridine, terpyridine, and phenanthroline pyrazole linkers. Functioning as a multiple N-based linker, the imidazo[1,5-*a*]pyridine skeleton can coordinate different metal ions, producing a wide array of coordination motifs and geometries. Its coordination chemistry includes bidentate or tridentate chelating systems which have been employed in the formation of metal complexes for different applications. Moreover, multitopic imidazo[1,5-*a*]pyridines have been easily synthesized in high yields with different linkers; this is a crucial structural aspect that ensures geometry tunability, equivalent to that observed for the most investigated polypyridines or polypyrazoles which are widely employed in coordination chemistry [33,34,35]. Finally, while elaborate ligands can be designed to impart specific properties, scalability will prove challenging if these are challenging to synthesize. Among the many promising imidazo[1,5-*a*]pyridine candidates, we selected the 1,4-bis(1-(pyridin-2-yl)imidazo[1,5-*a*]pyridin-3-yl)benzene (L) because this molecule has an ideal linear ditopic structure (Scheme 1) containing two chelating subunits (namely pyridinyl imidazo[1,5-*a*]pyridine), each based on the synergic combination of a pendant pyridine substituent on an imidazo[1,5-*a*]pyridine moiety. The chelation capability is ensured by the optimal bite angle between the pyridine and the imidazole nitrogen atoms. Furthermore, this molecule can be easily synthesized in high yield, through an efficient one-step double cyclization process, avoiding the use of any expensive metal catalysts, toxic reagents, or derivatives [33].

Summarizing, the following study reports on the first example of three Zn(II) coordination polymers incorporating the ditopic chelating pyridinyl imidazo[1,5-*a*]pyridine ligand and different dicarboxylic acid derivatives, namely the fumarate (fum) and the terephthalate (tpt) anions (Scheme 1). In particular, one polymer is formed with fumarate and L (hereafter referred to as **1**) and two polymers with terephthalate and L (hereafter referred to as **2** and **3**, with **2** being an aquo complex). The three new compounds have been synthetized, structurally characterized, and computationally analyzed to enhance the comprehension of the weak interactions that determine their arrangement in the crystals.

## 2. Results

### 2.1. Synthesis and Structural Description of Compounds ***1***–***3***

The aim of the study is to prove the concept that it is possible to obtain new coordination polymers having imidazo[1,5-*a*]pyridine moieties incorporated in the framework of Zn(II)-metallic nodes. To achieve this goal, the synthetic strategy was focused on obtaining crystals suitable for structural studies to evaluate the molecular assembly. For this purpose, we adopted a conventional synthesis method based on a series of non-solvothermal reactions, where small batches were prepared to grow some good quality crystals (refer to the syntheses methods section for details). This approach allowed us to obtain and characterize three new coordination polymers (**1**, **2**, and **3**).

**Figure 1:**
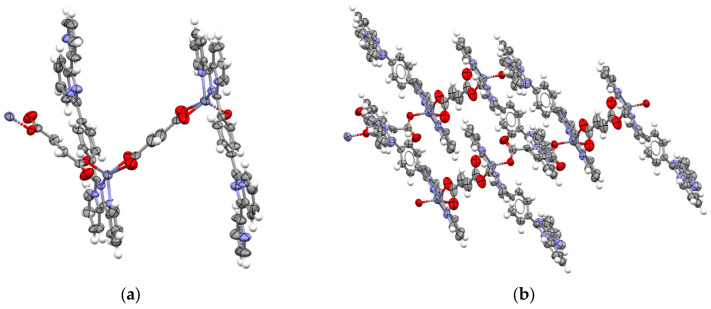
(**a**) Fragment of the 1D coordination polymer **1**; (**b**) crystal packing between ribbons in **1**. Color code: dark grey, zinc; grey, carbon; white, hydrogen; red, oxygen; violet, nitrogen (thermal ellipsoids drawn at 70% of probability).

The first coordination polymer (**1**) employs the fumarate anion as the linker between the Zn(II) centers in a 1D pattern, while the pyridinyl imidazo[1,5-*a*]pyridine derivative L has the role of ancillary ligand. The polymers crystallize in the triclinic space group as yellow prisms, stable to both air and moisture. The asymmetric unit contains one Zn(II) ion, one L, and two half fumarate fragments. The Zn(II) ion presents a typical tetrahedral coordination environment, which consists of two carboxylate ions from two fumarate molecules and two nitrogen sites of a chelating ligand L (see Figure 1). The two carboxylates are non-equivalent because one interacts with both the oxygen atoms in a chelating mode (d(O–Zn) = 2.530(8) Å and 1.981(8) Å), while the second is monodentate (d(O–Zn) = 2.973(8) Å and 1.941(8) Å). The two non-equivalent fumarate anions in the asymmetric unit lie on two inversion centers that generate the entire molecules. The anionic fum ligands with the metal centers generate a 1D wavy coordination polymer (see Figure 1a). In the ancillary ligand L, both pyridinyl imidazo[1,5-*a*]pyridine moieties (the chelating portion and the uncoordinated one) are planar, while the central phenyl ring is rotated (ang_tor_(N3–C12–C13–C15) = 38.17(20)°, ang_tor_(N6–C19–C18–C17) = 32.02(20)°). In the uncoordinated fragment, the pyridine N atom is opposite with respect to the imidazo[1,5-*a*]pyridine N atom, similarly to the two polymorphs of the free ligand [36]. All the aromatic systems of L are involved in strong π···π interactions that are commented on in depth in the following computational part (see below). These interactions involve the L of different chains in an interdigitated manner. The resulting packing imposes a parallel propagation of the Zn(II)-fum polymer chains (see Figure 1b).

**Figure 2:**
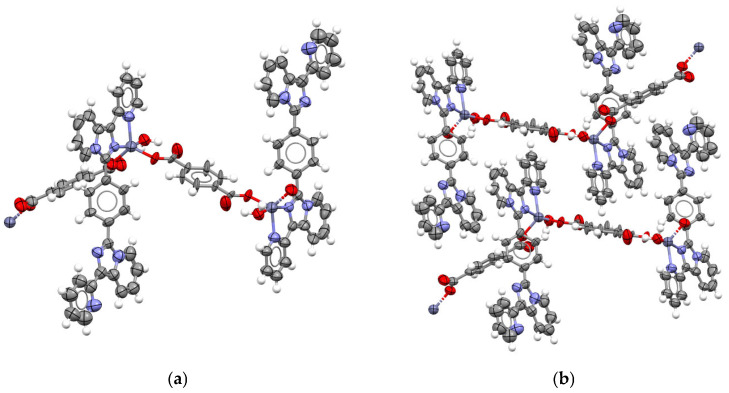
(**a**) Fragment of the 1D coordination polymer **2**; (**b**) crystal packing between ribbons in **2**. Color code: dark grey, zinc; grey, carbon; white, hydrogen; red, oxygen; violet, nitrogen (thermal ellipsoids drawn at 70% of probability).

The second coordination polymer (**2**) features the terephthalate anion as the linking unit between the Zn(II) centers in a 1D pattern, while, also in this case, L has the role of ancillary ligand. Also, these polymers crystallize in the triclinic space group as yellow prisms, stable in air and moisture. The asymmetric unit contains one Zn(II) ion, one L, two half terephthalate fragments, and one water molecule. In the structure of **2**, the Zn(II) ion is involved in a bipyramidal trigonal coordination environment which comprises two carboxylates, two chelating nitrogen atom sites of L, and a water molecule (refer to Figure 2). The two carboxylates, although independent, are more similar each other when compared to the structure of **1**; indeed, both interact in a monodentate fashion, employing only one oxygen atom (i.e., d(O–Zn) = 1.959(5) Å and 3.233(8) Å, d(O–Zn) = 1.977(5) Å and 3.346(8) Å). The other oxygen atom is involved in an O–H⋅⋅⋅O hydrogen bond with the water ligand (d(O–O) = 2.626(6) Å). The entire terephthalate moieties are generated by a central inversion center and form a 1D wavy coordination polymer along with the metal centers (see Figure 2a). Similarly to **1**, the pyridinyl imidazo[1,5-*a*]pyridine moieties of L are planar, while the central phenyl ring is tilted (ang_tor_(N3–C20–C21–C22) = 39.47(20)°, ang_tor_(N4–C27–C24–C23) = 34.16(20)°) and, like happens for **1**, only one pyridinyl imidazo[1,5-*a*]pyridine coordinates the Zn(II) ion, while in the other, the two chelating N atoms are oriented oppositely, resembling the free L. All the aromatic systems are involved in strong π···π interactions that are commented on in depth in the computational part (see below). As in **1**, the resulting packing is characterized by the parallel propagation of the Zn(II)-tpt polymer chains (see Figure 2b) linked by the π···π interactions between L of different chains in an interdigitated manner.

**Figure 3:**
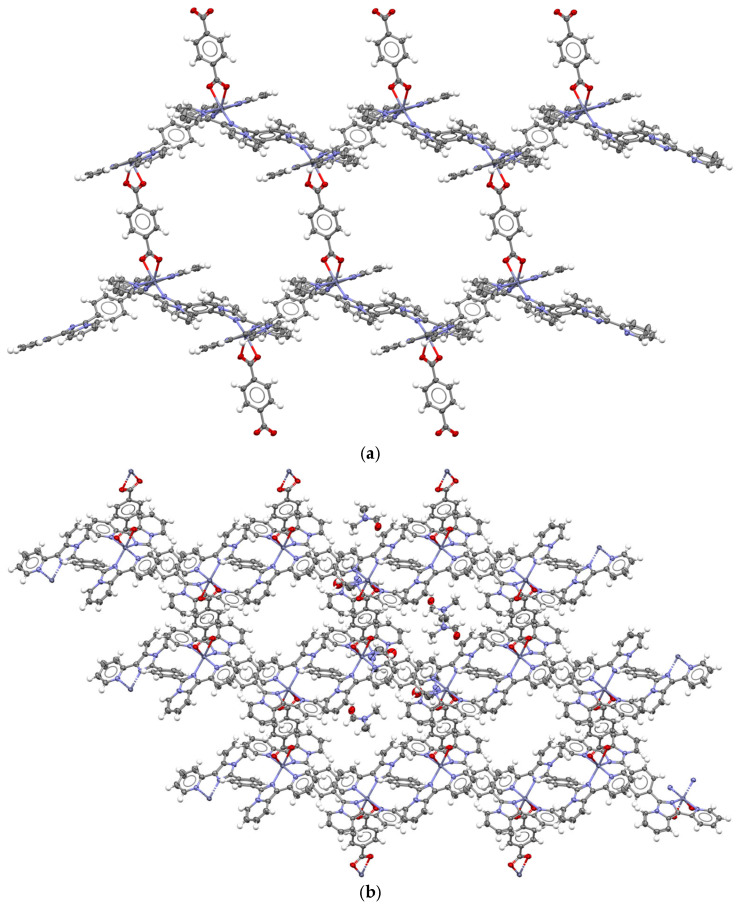
(**a**) Fragment of the 2D coordination polymer **3**; (**b**) channels in **3**. Color code: dark grey, zinc; grey, carbon; white, hydrogen; red, oxygen; violet, nitrogen (thermal ellipsoids drawn at 70% of probability).

The third coordination polymer (**3**) differs significantly from **1** and even from **2**, despite sharing the same ligands. Specifically, **3** presents the terephthalate anion as a link between the Zn(II) centers but adopts a 2D pattern, where the pyridinyl imidazo[1,5-*a*]pyridine derivative L also acts as a connecting ligand. The **3** polymers crystallize in the triclinic space group as yellow prisms that are sensitive to air, probably due to the loss of the structural DMF; so, the measurements were performed at a low temperature. The asymmetric unit is characterized by one Zn(II) ion, two half L fragments, one half tpt fragment, and two DMF molecules. The Zn(II) ions present a bipyramidal trigonal coordination environment with one carboxylate and four nitrogen sites of two L (see Figure 3a). The terephthalate anions and the two non-equivalent L are generated by three inversion centers. In this case, the tpt coordinates the Zn(II) ions with both oxygen atoms (d(O–Zn) = 2.223(5) Å and 2.272(5) Å). The chelating parts of the two ligands L are planar due to geometric constraints imposed by complexation, while the central phenyl rings are rotated: one with torsional angles of 50.31(20)° and the other with torsional angles of 44.82(20)°. Also in this case, all the aromatic portions are involved in strong π···π interactions, extensively commented on in the computational section (see below). The pattern of L and tpt with the zinc nodes form a (4,4) net with open spaces. In the resulting channels, two DMF molecules lie without any interactions (see Figure 3b). However, they significantly contribute to the stability of the crystal since the sample is stable at room temperature only for a few hours if not kept in the DMF mother liquor. Presumably, the structure collapses when the crystal begins to lose DMF molecules.

### 2.2. Theoretical Study

First and foremost, compounds **1**–**3** are coordination polymers and, as previously discussed, their solid-state structures are predominantly defined by coordination bonds. This is particularly evident in compound **3,** which shows a 2D coordination network. On the other hand, compounds **1** and **2**, whose main motifs are chains, also exhibit significant π-stacking interactions and hydrogen bonding in their crystal packing to maintain the integrity of the chains. To assess the energetics of these π-stacking and hydrogen bonds in compounds **1** and **2**, we employed DFT calculations alongside QTAIM and NCIplot analyses (refer to the computational methods section below for details).

**Scheme 2:**
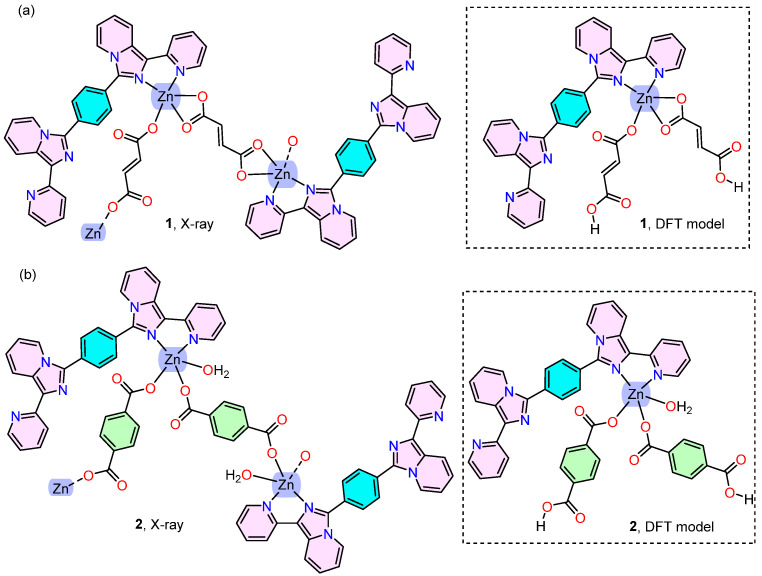
(**a**) Chemical diagram of polymer **1** (**left**) and the simplified monomeric model used in this work (**right**). (**b**) chemical diagram of polymer **2** (**left**) and the simplified monomeric model used in this work (**right**).

Given the polymeric nature of these compounds (see Scheme 2a,b, left), we utilized a monomeric fragment of each for evaluating π-stacking interactions. Specifically, for compound **1**, the fumarate ligands were protonated at one end to yield a neutral [ZnL(Hfum)_2_] mononuclear complex (see Scheme 2a, right). Likewise, for compound **2**, the bridging terephthalate ligands underwent protonation, resulting in the [ZnL(H_2_O)(Htpt)_2_] monomeric complex (refer to Scheme 2b, right).

**Figure 4:**
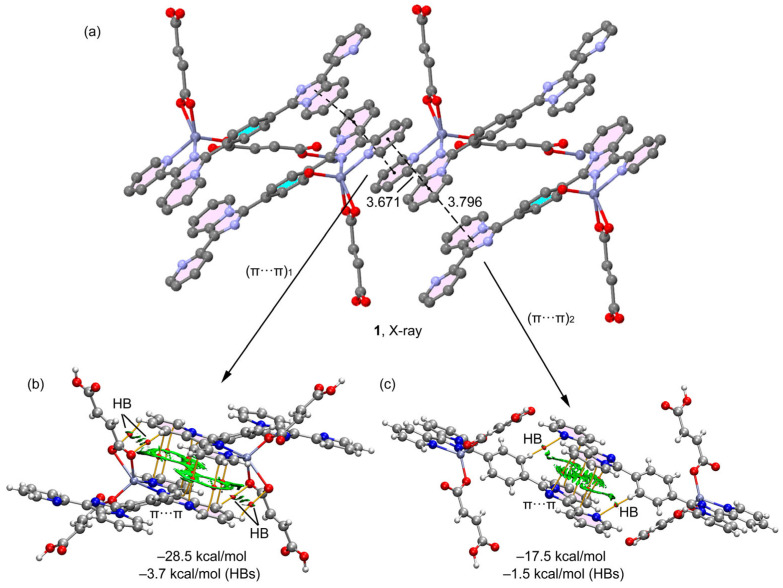
(**a**) Partial view of the X-ray structure of compound **1**. (**b**) QTAIM and NCIplot analysis of (π···π)_1_ assembly, only intermolecular interactions are shown. (**c**) QTAIM and NCIplot analysis of (π···π)_2_ assembly, only intermolecular interactions are shown.

Using these models, we examined and compared the π-stacking assemblies identified in the X-ray packing of compounds **1** and **2**. Figure 4a provides a partial view of the X-ray structure of compound **1**, highlighting the formation of 1D chains interconnected by two distinct types of π-stacking interactions, labeled as (π···π)_1_ and (π···π)_2_ in Figure 4. Both interactions were energetically evaluated using this simplified theoretical model and characterized via the quantum theory of atoms-in-molecules (QTAIM) and the noncovalent interaction plot (NCIPlot). The combined QTAIM/NCIPlot is advantageous for visualizing noncovalent interactions in real space. Notably, in the (π···π)_1_ stacked dimer, the monomers are linked through numerous bond critical points (BCPs) and bond paths. Specifically, each coordinated pyridine ring from one molecule connects to the uncoordinated pyridine ring of the neighboring molecule via four BCPs and bond paths (and vice versa). Additionally, extensive and green reduced density gradient (RDG) isosurfaces manifest between the π-systems, indicating a robust overlap of the π-systems. Intriguingly, the QTAIM/NCIPlot analysis also unveils CH···O contacts between aromatic CH bonds and both O-atoms of the coordinated fumarate ligands. Each CH···O contact is distinguished by its respective BCP, bond path, and a compact green RDG isosurface.

For the (π···π)_2_ stacked dimer illustrated in Figure 4c, π-stacking is characterized by five BCPs and bond paths linking the six-membered and five-membered rings of the imidazo[1,5-*a*]pyridine unit. A substantial green RDG isosurface between the π-systems further demonstrates the complementarity of these systems. The QTAIM/NCIPlot analysis uncovers two symmetrically equivalent CH···N contacts between the aromatic CH bonds of the phenyl rings and the N-atoms of the uncoordinated pyridine rings. Each of these CH···N contacts is characterized by its respective BCP, bond path, and a small green RDG isosurface, as illustrated in Figure 4c. Both π-stacking interactions hold significant energetic importance, with dimerization energies of –28.5 kcal/mol for (π···π)_1_ and –17.5 kcal/mol for (π···π)_2_. The superior binding energy of the (π···π)_1_ stacking mode aligns well with its more extensive RDG isosurface. By utilizing potential energy density values at the BCPs, we estimated the contributions from CH···O and CH···N hydrogen bonds (see computational methods below). As detailed in Figure 4, these contributions are minimal compared to the overall interaction energies: –3.7 and –1.5 kcal/mol for (π···π)_1_ and (π···π)_2_, respectively. This indicates that dimer formation is overwhelmingly driven by π-stacking.

**Figure 5:**
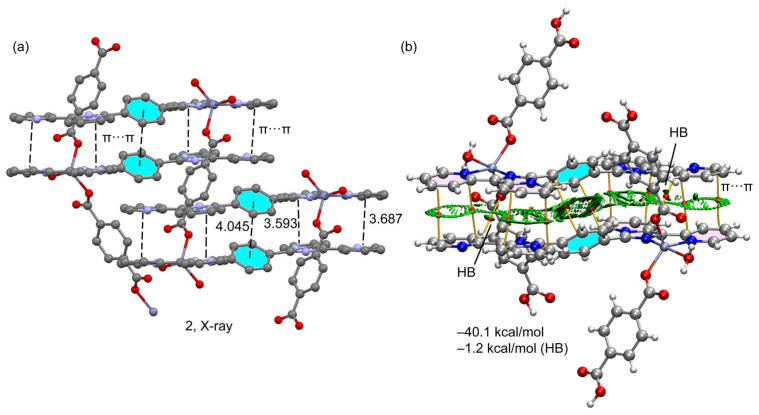
(**a**) Partial view of the X-ray structure of compound **2**. (**b**) QTAIM and NCIplot analysis of π···π assembly, only intermolecular interactions are shown.

In compound **2**, which incorporates the terephthalate linker, a distinct stacking mode is evident (refer to Figure 5a) involving the π-system of the entire moiety. The combined QTAIM/NCIplot analysis reveals ten BCPs and bond paths connecting the monomers, accompanied by an extensive and continuous RDG isosurface. Mirroring the dimers in compound **1**, two additional CH···O contacts are apparent, as denoted by the BCPs and bond paths linking the H and O-atoms. This assembly possesses a substantial interaction energy of –40.1 kcal/mol, attributed to the involvement of all the seven aromatic rings of the imidazo[1,5-*a*]pyridine unit. This pronounced interaction energy underscores the crucial role of π-stacking in shaping the solid-state structure of compound **2**, complementing the coordination bonds. The CH···O contacts contribute a negligible amount (–1.2 kcal/mol) when juxtaposed with the π-stacking interaction. The large π-stacking energies observed in compounds **1** and **2** arise from the antiparallel orientation of the π-systems, synergized with metal coordination. This coordination bolsters dipole–dipole attraction, a phenomenon previously documented in the literature [37,38].

## 3. Materials and Methods

### 3.1. Experimental Techniques

All solvents and raw materials were used as received from commercial suppliers (Sigma-Aldrich, Saint Louis, MO, USA, and Alfa Aesar, Ward Hill, MA, USA) without further purification.

Single crystal data of the crystallized compounds **1**, **2** and **3** were collected on a Gemini R Ultra diffractometer (Agilent Technologies UK Ltd., Oxford, UK) using graphite-monochromatic Cu Kα radiation (λ = 1.5406 Å) with the ω-scan method. Copper-derived radiation was preferred for the cases of very weakly diffracting crystals. The CrysAlisPro V42 software was used for retrieving cell parameters, for performing data reduction, and for absorption correction (with multi-scan technique). All the structures were solved by direct methods using ShelXS-14 [39] and refined with full-matrix least-squares on F^2^ with SHELXL-14 [40] using Olex^2^ program [41]. All non-hydrogen atoms were anisotropically refined. Hydrogen atoms were inserted in calculated positions and refined as riding on the corresponding atom.

The images of the structures were generated using the software Mercury 2023.1.0, developed by the Cambridge Crystallographic Data Centre [42]. All the crystal data and refinement, selected bond lengths and angle amplitudes, and asymmetric units of compounds are reported in the Supplementary Materials section (Tables S1–S9, Figures S1–S3). The crystallographic data for the compounds are deposited within the Cambridge Crystallographic Data Centre as supplementary publications under the CCDC numbers 2320594, 2320595, and 2320596. This information can be obtained free of charge from the Cambridge Crystallographic Data Centre.

### 3.2. Syntheses

The ligand 1,4-bis(1-(pyridin-2-yl)imidazo[1,5-*a*]pyridin-3-yl)benzene (L) was prepared as previously reported [33].

The three coordination polymers (**1**–**3**) were obtained following a conventional synthesis method, developed using a series of non-solvothermal reactions [43]. The experimental studies were focused on obtaining crystals suitable for structural studies to evaluate the molecular assembly. To achieve this goal, small batches were prepared to grow a limited number of high-quality crystals. In order to enhance the crystal quality, various combinations of parameters were tested. According to the literature, the temperature has the strongest influence on the crystallization process and strongly impacts the crystal morphology [44]. The experimental protocol consisted of crystallizations performed at different temperatures, particularly exploring the range between 4 °C and 25 °C. The best results were achieved performing the crystallizations at 4 °C under atmospheric pressure. However, the overall process is also influenced by other parameters, and the crystal growth can be optimized by adjusting the reaction conditions and/or by changing parameters like the concentration of the solutions of the starting raw materials, evaporation rates, and diffusion rates.

The first coordination polymer (**1**) is characterized by the presence of the fumarate anion as a linker and was obtained by dissolving the three components (L, Zn(NO_3_)_2_, and fumaric acid) in 5 mL of dimethylformamide (DMF) at a stoichiometric ratio of 1:2:1, respectively (20.00 mg of L, 18.98 mg of Zn(NO_3_)_2_, 5.00 mg of fumaric acid). To promote the formation of crystals, 1 mL of ethyl ether was layered over the DMF solution. The evaporation rate was controlled by maintaining the temperature at 4 °C.

The other two coordination polymers (compound **2** and **3**) are characterized by the presence of the terephthalate anion as a linker. They were obtained using two distinct approaches and two different concentration ratios.

For the synthesis of **2**, an overall stoichiometric ratio of 1:1:1 (L, Zn(NO_3_)_2_, and terephthalic acid) was employed. A first solution was prepared by adding Zn(NO_3_)_2_ (15.18 mg) and terephthalic acid (22.93 mg) to 5 mL of DMF. Subsequently, a solution of L (32.00 mg) in 1 mL of dichloromethane was layered over the DMF solution containing the Zn(II) salt and the organic linker. The evaporation rate was controlled by fixing the temperature at 4 °C.

Compound **3** was prepared by dissolving all the three reagents (32.00 mg of L, 15.18 mg of Zn(NO_3_)_2_, and 22.93 mg of terephthalic acid) in 5 mL of DMF, using a stoichiometric ratio of 1:2:1, and layering 1 mL of ethyl ether over the DMF solution. The evaporation rate was controlled by fixing the temperature at 4 °C.

All the single crystals obtained were stored at –40.0 °C to prevent complete solvent evaporation. Different stoichiometries were attempted in all syntheses, resulting in the reported compounds, along with an excess of unreacted ligand, either L or fum or tpt, depending on the stoichiometry.

### 3.3. Theoretical Methods

In this study, we computed the energies and wave-functions of the examined compounds and supramolecular assemblies utilizing the RI-BPE0-D4/def2-TZVP theoretical level [45,46] with X-ray coordinates, employing the Turbomole 7.7 program [47]. We incorporated Grimme’s D4 dispersion correction [48], which is particularly adept at accurately evaluating non-covalent interactions, especially those associated with π-systems such the ones examined in this study. For characterizing non-covalent interactions, we employed Bader’s QTAIM method [49], alongside the NCI plot [50] reduced density gradient (RGD) isosurfaces. These tools are instrumental in visually representing non-covalent interactions within a real space context. The cubes required for creating the NCI plot surfaces were determined at the same theoretical level, utilizing the wave functions generated by the Turbomole 7.7 software. It is important to note that the NCI plot index isosurfaces represent both favorable and unfavorable interactions, distinguishable by the sign of the second density Hessian eigenvalue and highlighted through the isosurface color. Finally, the QTAIM analysis was conducted at the same theoretical level using the MULTIWFN program [51] and visualized through the software VMD 1.9 [52]. The formation energies of the hydrogen bonds were estimated by using the value of the potential energy density at the BCPs, employing the equation proposed by Espinosa et al. (E = 0.5 × Vr) [53].

## 4. Conclusions

This manuscript reports on the synthesis and the X-ray characterization of three new Zn(II) coordination polymers, **1**–**3**, obtained employing a promising ditopic etherocyclic ligand L, which is based on the pyridinyl imidazo[1,5-*a*]pyridine skeleton, and dicarboxylic acid derivatives, namely terephthalate and fumarate anions. The X-ray structures were solved highlighting monodimensional motifs (chains) for **1** and **2** and a 2D network for **3**.

The theoretical investigation of compounds **1** and **2** underscores the significant role of π-stacking interactions and their profound impact on the solid-state structures of coordination polymers. The rigorous QTAIM/NCIplot analysis evidences the presence of numerous BCPs and bond paths, indicating the interplay of aromatic systems within these compounds. Notably, while additional CH···O and CH···N contacts are identified, their energetic contributions are markedly overshadowed by the dominant π-stacking interactions. In the case of compound **2**, the comprehensive involvement of the π-system of the terephthalate linker further accentuates the role of π-stacking in the solid-state architecture, complementing the coordination bonds.

In general, the work provides an important insight into the design of new coordination polymers, thanks to the versatile skeleton of pyridinyl imidazo[1,5-*a*]pyridine derivatives. The work demonstrates the strong affinity of the ligand L towards Zn(II) metal nodes. Moreover, its structural versatility admits multitopic chelating coordination geometries, among the various families of aromatic heterocyclic compounds, and imidazo[1,5-*a*]pyridine derivatives can be easily obtained in high yields through one-pot synthetic approaches, as extensively demonstrated in the literature [33,34,54].

The study demonstrates the applicability of versatile imidazo[1,5-*a*]pyridine ligands to assemble new Zn(II)-based coordination polymers. In particular, this work highlights the significance of π-stacking interactions in the crystal packing of coordination polymers, offering valuable insights that may guide future design and synthesis efforts in the realms of coordination networks and supramolecular chemistry. A future objective is to scale up the synthesis and optimize the process to obtain these materials in bulk with good yield.

## Data Availability

The data presented in this study are available within the article and the Supplementary Materials.

## Supplementary Materials

The following supporting information can be downloaded at: https://www.mdpi.com/article/10.3390/molecules29030653/s1, Figure S1: ORTEP plot of the asymmetric unit of **1**; Table S1: Crystal data and structure refinement for **1**; Table S2: Bond lengths for **1**; Table S3: Bond angles for **1**; Figure S2: ORTEP plot of the asymmetric unit of **2**; Table S4: Crystal data and structure refinement for **2**; Table S5: Bond lengths for **2**; Table S6: Bond angles for **2**; Figure S3: ORTEP plot of the asymmetric unit of **3**; Table S7: Crystal data and structure refinement for **3**; Table S8: Bond lengths for **3**; Table S9: Bond angles for **3**.
