# Supplementary material for: One-Dimensional and Two-Dimensional Zn(II) Coordination Polymers with Ditopic Imidazo[1,5-a]pyridine: A Structural and Computational Study
Source: Molecules. 2024 Jan 30;29(3):653. doi: 10.3390/molecules29030653 (PMC10856496; doi:10.3390/molecules29030653)
Supplement: Supplementary file 1 [file molecules-29-00653-s001.zip › molecules-2828393-supplementary.pdf]

One-Dimensional and Two-Dimensional Zn(II) Coordination Polymers with Ditopic Imidazo[1,5-a]pyridine: A Structural and Computational Study

Mattia Sozzi [1], Michele R. Chierotti [1], Roberto Gobetto [1], Rosa M. Gomila [2], Vittoria Marzaroli [1], Emanuele Priola [1], Giorgio Volpi [1], Stefano Zago [1], Antonio Frontera [2,*] and Claudio Garino [1,*]

1 Department of Chemistry and NIS Centre, University of Turin, Via P. Giuria, 7 - 10125 Turin, Italy
2 Department of Chemistry, Universitat de les Illes Balears, Crta de Valldemossa km 7.5, 07122 Palma de Mallorca, Spain
E-mail: claudio.garino@unito.it, toni.frontera@uib.es

Figure S1. ORTEP plot of the asymmetric unit of [Zn(fum)(L)] (**1**). 2
Table S1. Crystal data and structure refinement for [Zn(fum)(L)] (**1**). 2
Table S2. Bond lengths for [Zn(fum)(L)] (**1**). 3
Table S3. Bond angles for [Zn(fum)(L)] (**1**). 4
Figure S2. ORTEP plot of the asymmetric unit of [Zn(tpt)(L)(H_2O)] (**2**). 5
Table S4. Crystal data and structure refinement for [Zn(tpt)(L)(H_2O)] (**2**). 5
Table S5. Bond lengths for [Zn(tpt)(L)(H_2O)] (**2**). 6
Table S6. Bond angles for [Zn(tpt)(L)(H_2O)] (**2**). 7
Figure S3. ORTEP plot of the asymmetric unit of [Zn(tpt)$(L)_2$]·2DMF (**3**). 8
Table S7. Crystal data and structure refinement for [Zn(tpt)$(L)_2$]·2DMF (**3**). 8
Table S8. Bond lengths for [Zn(tpt)$(L)_2$]·2DMF (**3**). 9
Table S9. Bond angles for [Zn(tpt)$(L)_2$]·2DMF (**3**). 9

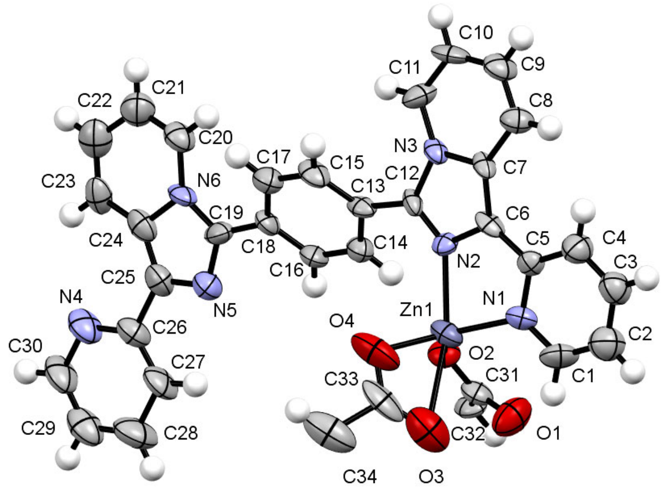

Figure S1. ORTEP plot of the asymmetric unit of [Zn(fum)(L)] (**1**).

Table S1. Crystal data and structure refinement for [Zn(fum)(L)] (**1**).

Empirical formula	$C_{34}H_{22}N_6O_4Zn$
Formula weight	643.94
Temperature/K	293.00
Crystal system	triclinic
Space group	P-1
a/Å	10.547(6)
b/Å	11.969(7)
c/Å	13.786(8)
α/°	64.879(9)
β/°	79.643(11)
γ/°	70.890(10)
Volume/Å^3	1487.2(15)
Z	2
ρ_{calc}g/cm^3	1.438
μ/mm^{-1}	0.876
F(000)	660.0
Crystal size/mm^3	0.13 × 0.11 × 0.1
Radiation	MoKα (λ = 0.71073)
2Θ range for data collection/	3.266 to 54.576
Index ranges	-9 ≤ h ≤ 13, -11 ≤ k ≤ 15, -12 ≤ l ≤ 17
Reflections collected	11310
Independent reflections	6690 [R_{int} = 0.1235, R_{sigma} = 0.0970]
Data/restraints/parameters	6690/0/406
Goodness-of-fit on F^2	0.991
Final R indexes [I>=2σ (I)]	R_1 = 0.0530, wR_2 = 0.1067
Final R indexes [all data]	R_1 = 0.1035, wR_2 = 0.1255
Largest diff. peak/hole / e Å^{-3}	0.28/-0.29

Table S2. Bond lengths for [Zn(fum)(L)] (**1**).

Atom	Atom	Length/Å		Atom	Atom	Length/Å
Zn1	O2	1.915(6)		C15	C13	1.382(11)
Zn1	N2	2.117(7)		C8	C9	1.328(11)
Zn1	N1	2.054(7)		C19	N5	1.333(10)
Zn1	O4	1.981(8)		C19	C18	1.479(12)
Zn1	C33	2.497(13)		C5	C6	1.426(11)
C20	N6	1.376(11)		C5	C4	1.370(11)
C20	C21	1.337(11)		N5	C25	1.383(11)
N3	C7	1.376(9)		C14	C13	1.395(10)
N3	C12	1.399(10)		C14	C16	1.366(10)
N3	C11	1.379(10)		C11	C10	1.344(11)
O2	C31	1.284(10)		C18	C16	1.361(10)
N6	C24	1.440(10)		O4	C33	1.204(14)
N6	C19	1.348(10)		C26	C27	1.363(12)
N2	C12	1.306(9)		C26	C25	1.488(13)
N2	C6	1.367(9)		C10	C9	1.416(11)
N1	C5	1.360(10)		C31	C32	1.501(12)
N1	C1	1.322(10)		C4	C3	1.347(11)
O3	C33	1.268(14)		C23	C22	1.354(12)
O1	C31	1.212(10)		C22	C21	1.434(12)
C7	C8	1.435(11)		C29	C28	1.370(13)
C7	C6	1.401(11)		C29	C30	1.367(13)
N4	C26	1.330(11)		C27	C28	1.427(12)
N4	C30	1.361(12)		C3	C2	1.374(12)
C24	C23	1.393(12)		C2	C1	1.362(12)
C24	C25	1.364(13)		C33	C34	1.585(17)
C12	C13	1.457(11)		C32	C32[1]	1.288(15)
C17	C15	1.392(11)		C34	C34[2]	1.25(2)
C17	C18	1.387(11)				

[1]1-X,1-Y,1-Z; [2]1-X,-Y,2-Z

Table S3. Bond angles for [Zn(fum)(L)] (**1**).

Atom	Atom	Atom	Angle/°		Atom	Atom	Atom	Angle/°
O2	Zn1	N2	106.5(3)		C16	C14	C13	121.3(8)
O2	Zn1	N1	122.1(3)		C10	C11	N3	119.0(9)
O2	Zn1	O4	112.4(3)		N2	C6	C7	105.7(8)
O2	Zn1	C33	106.5(3)		N2	C6	C5	119.7(9)
N2	Zn1	C33	127.5(4)		C7	C6	C5	134.6(10)
N1	Zn1	N2	79.6(4)		C17	C18	C19	123.2(10)
N1	Zn1	C33	114.2(4)		C16	C18	C17	117.1(8)
O4	Zn1	N2	100.5(3)		C16	C18	C19	119.7(10)
O4	Zn1	N1	123.2(3)		C15	C13	C12	126.2(9)
O4	Zn1	C33	28.3(4)		C15	C13	C14	116.1(8)
C21	C20	N6	120.9(9)		C14	C13	C12	117.7(9)
C7	N3	C12	105.5(8)		C33	O4	Zn1	100.4(9)
C7	N3	C11	122.3(8)		N4	C26	C27	123.5(9)
C11	N3	C12	132.2(10)		N4	C26	C25	116.8(12)
C31	O2	Zn1	119.0(6)		C27	C26	C25	119.6(12)
C20	N6	C24	118.7(9)		C11	C10	C9	120.2(9)
C19	N6	C20	133.6(10)		O2	C31	C32	114.8(9)
C19	N6	C24	107.5(9)		O1	C31	O2	124.3(9)
C12	N2	Zn1	139.0(7)		O1	C31	C32	120.9(10)
C12	N2	C6	110.5(8)		C3	C4	C5	120.7(9)
C6	N2	Zn1	110.4(7)		C22	C23	C24	121.2(10)
C5	N1	Zn1	115.3(7)		C18	C16	C14	122.9(9)
C1	N1	Zn1	126.2(8)		C23	C22	C21	118.4(10)
C1	N1	C5	118.5(8)		C30	C29	C28	120.2(11)
N3	C7	C8	117.7(9)		C8	C9	C10	121.3(9)
N3	C7	C6	108.7(8)		C26	C27	C28	119.8(10)
C6	C7	C8	133.6(11)		C4	C3	C2	119.6(10)
C26	N4	C30	116.5(10)		C20	C21	C22	121.6(10)
C23	C24	N6	119.0(11)		C29	C28	C27	116.3(11)
C25	C24	N6	103.0(10)		C1	C2	C3	117.7(10)
C25	C24	C23	138.0(12)		N1	C1	C2	123.5(10)
N3	C12	C13	123.8(10)		N4	C30	C29	123.5(11)
N2	C12	N3	109.6(8)		O3	C33	Zn1	77.2(8)
N2	C12	C13	126.4(9)		O3	C33	C34	117.4(14)
C18	C17	C15	120.5(9)		O4	C33	Zn1	51.3(7)
C13	C15	C17	122.1(8)		O4	C33	O3	128.1(14)
C9	C8	C7	119.4(9)		O4	C33	C34	114.5(14)
N6	C19	C18	127.8(10)		C34	C33	Zn1	164.4(12)
N5	C19	N6	112.0(9)		C32[1]	C32	C31	126.6(11)
N5	C19	C18	120.2(10)		C34[2]	C34	C33	125.7(19)
N1	C5	C6	114.7(9)		C24	C25	N5	112.6(9)
N1	C5	C4	119.8(9)		C24	C25	C26	129.2(12)
C4	C5	C6	125.5(10)		N5	C25	C26	118.2(12)
C19	N5	C25	104.9(8)					

[1]1-X,1-Y,1-Z; [2]1-X,-Y,2-Z

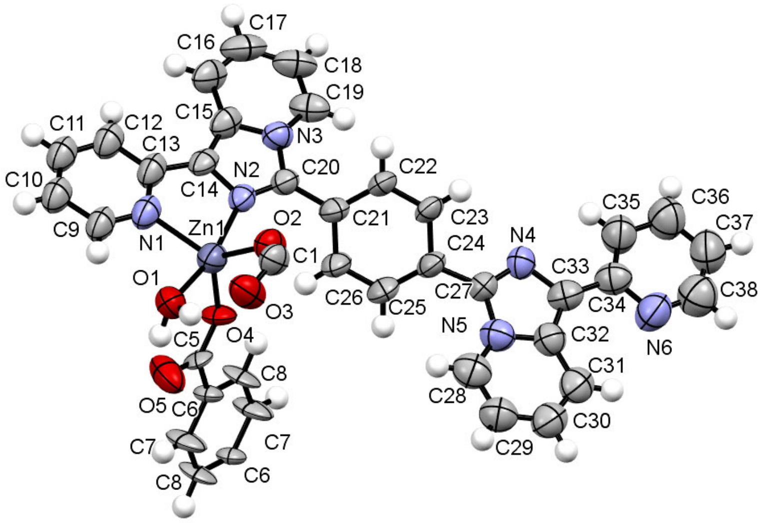

Figure S2. ORTEP plot of the asymmetric unit of [Zn(tpt)(L)(H_2O)] (**2**).

Table S4. Crystal data and structure refinement for [Zn(tpt)(L)(H_2O)] (**2**).

Empirical formula	$C_{38}H_{26}N_6O_5Zn$
Formula weight	712.02
Temperature/K	298.0
Crystal system	triclinic
Space group	P-1
a/Å	11.9196(16)
b/Å	12.271(2)
c/Å	13.308(2)
α/°	75.124(14)
β/°	87.043(12)
γ/°	82.912(12)
Volume/Å^3	1866.6(5)
Z	2
ρ_{calc}g/cm^3	1.267
μ/mm^{-1}	1.316
F(000)	732.0
Crystal size/mm^3	0.21 × 0.18 × 0.15
Radiation	CuKα (λ = 1.54184)
2Θ range for data collection/°	6.874 to 135.054
Index ranges	-8 ≤ h ≤ 14, -8 ≤ k ≤ 14, -8 ≤ l ≤ 15
Reflections collected	6720
Independent reflections	6420 [R_{int} = 0.0634, R_{sigma} = 0.0744]
Data/restraints/parameters	6420/437/452
Goodness-of-fit on F^2	1.088
Final R indexes [I>=2σ (I)]	R_1 = 0.0549, wR_2 = 0.1420
Final R indexes [all data]	R_1 = 0.0726, wR_2 = 0.1699
Largest diff. peak/hole / e Å^{-3}	0.25/-0.25

Table S5. Bond lengths for [Zn(tpt)(L)(H_2O)] (**2**).

Atom	Atom	Length/Å		Atom	Atom	Length/Å
Zn1	O1	2.115(8)		C17	C18	1.40(2)
Zn1	N2	2.146(13)		C1	C2	1.53(2)
Zn1	O2	1.978(10)		C2	C3	1.361(17)
Zn1	O4	1.959(10)		C2	C4[2]	1.377(16)
Zn1	N1	2.085(13)		C24	C25	1.406(19)
N2	C14	1.388(17)		C24	C23	1.422(19)
N2	C20	1.299(17)		C24	C27	1.46(2)
O2	C1	1.265(17)		C25	C26	1.373(17)
O4	C5	1.258(18)		C26	C21	1.404(18)
O3	C1	1.246(17)		C21	C20	1.46(2)
C7	C6	1.34(2)		C9	C10	1.382(18)
C7	C8	1.376(16)		C3	C4	1.414(16)
C6	C5	1.46(2)		N5	C32	1.394(17)
C6	C8[1]	1.404(19)		N5	C28	1.357(16)
O5	C5	1.248(19)		N5	C27	1.402(17)
C22	C21	1.421(19)		N4	C33	1.419(17)
C22	C23	1.378(16)		N4	C27	1.291(16)
N3	C15	1.381(18)		C31	C32	1.442(19)
N3	C19	1.358(17)		C31	C30	1.351(17)
N3	C20	1.373(18)		C32	C33	1.368(19)
N1	C13	1.343(18)		C33	C34	1.415(19)
N1	C9	1.313(15)		C29	C28	1.389(18)
C14	C13	1.43(2)		C29	C30	1.373(18)
C14	C15	1.39(2)		N6	C34	1.358(18)
C12	C13	1.39(2)		N6	C38	1.317(18)
C12	C11	1.351(16)		C35	C34	1.345(18)
C16	C15	1.42(2)		C35	C36	1.383(18)
C16	C17	1.337(19)		C38	C37	1.35(2)
C11	C10	1.346(17)		C37	C36	1.385(19)
C19	C18	1.328(19)				

[1]1-X,1-Y,2-Z; [2]2-X,2-Y,1-Z

Table S6. Bond angles for [Zn(tpt)(L)(H_2O)] (**2**).

Atom	Atom	Atom	Angle/˚		Atom	Atom	Atom	Angle/˚
O1	Zn1	N2	166.4(4)		C3	C2	C1	120(2)
O2	Zn1	O1	92.2(4)		C3	C2	C4[2]	119.9(15)
O2	Zn1	N2	89.9(4)		C4[2]	C2	C1	121(2)
O2	Zn1	N1	127.9(4)		C25	C24	C23	116.2(19)
O4	Zn1	O1	94.6(4)		C25	C24	C27	128(2)
O4	Zn1	N2	96.2(4)		C23	C24	C27	116(2)
O4	Zn1	O2	118.5(4)		C26	C25	C24	123(2)
O4	Zn1	N1	113.2(4)		C25	C26	C21	120.8(19)
N1	Zn1	O1	89.7(4)		C22	C21	C20	121.9(18)
N1	Zn1	N2	78.5(5)		C26	C21	C22	116.6(17)
C14	N2	Zn1	110.4(12)		C26	C21	C20	121.5(19)
C20	N2	Zn1	141.9(15)		C22	C23	C24	120.5(19)
C20	N2	C14	107.5(19)		C7	C8	C6[1]	120.1(17)
C1	O2	Zn1	128.7(12)		N2	C20	N3	112(2)
C5	O4	Zn1	131.8(14)		N2	C20	C21	124(2)
C6	C7	C8	121.8(18)		N3	C20	C21	124(2)
C7	C6	C5	123(3)		N1	C9	C10	121.6(17)
C7	C6	C8[1]	118.1(16)		C11	C10	C9	117.9(18)
C8[1]	C6	C5	119(3)		C2	C3	C4	120.7(15)
C23	C22	C21	122.5(17)		C19	C18	C17	116(2)
C19	N3	C15	121.6(19)		C2[2]	C4	C3	119.5(15)
C19	N3	C20	132(2)		C32	N5	C27	102.8(18)
C20	N3	C15	106(2)		C28	N5	C32	124(2)
C13	N1	Zn1	116.6(14)		C28	N5	C27	133(2)
C9	N1	Zn1	122.8(12)		C27	N4	C33	107.6(18)
C9	N1	C13	120.6(18)		C30	C31	C32	117.5(19)
N2	C14	C13	119(2)		N5	C32	C31	116(2)
N2	C14	C15	108(2)		C33	C32	N5	111(2)
C15	C14	C13	133(2)		C33	C32	C31	133(2)
C11	C12	C13	118.4(19)		C32	C33	N4	106(2)
N1	C13	C14	115(2)		C32	C33	C34	134(2)
N1	C13	C12	119.7(19)		C34	C33	N4	120(2)
C12	C13	C14	125(2)		C30	C29	C28	117.2(18)
O4	C5	C6	120(2)		N5	C28	C29	119.4(19)
O5	C5	O4	121(2)		C38	N6	C34	112.6(19)
O5	C5	C6	119(2)		C34	C35	C36	122(2)
C17	C16	C15	120(2)		N6	C34	C33	113(2)
N3	C15	C14	107(2)		C35	C34	C33	124(2)
N3	C15	C16	116.0(19)		C35	C34	N6	123(2)
C14	C15	C16	137(2)		N5	C27	C24	124(2)
C10	C11	C12	121.7(19)		N4	C27	C24	123(2)
C18	C19	N3	123(2)		N4	C27	N5	113.1(18)
C16	C17	C18	123(2)		C31	C30	C29	125.4(19)
O2	C1	C2	113.1(19)		N6	C38	C37	129(2)
O3	C1	O2	129.1(18)		C38	C37	C36	117(2)
O3	C1	C2	118(2)		C35	C36	C37	115.4(19)

[1]1-X,1-Y,2-Z; [2]2-X,2-Y,1-Z

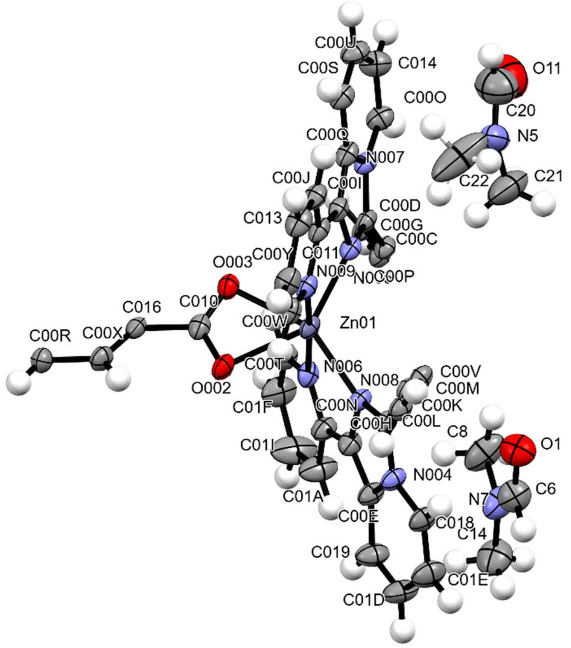

Figure S3. ORTEP plot of the asymmetric unit of [Zn(tpt)(L)$_2$]·2DMF (**3**).

Table S7. Crystal data and structure refinement for [Zn(tpt)(L)$_2$]·2DMF (**3**).

Empirical formula	$C_{40}H_{36}N_8O_4Zn$	
Formula weight	758.14	
Temperature/K	100.00	
Crystal system	triclinic	
Space group	P-1	
a/Å	12.376(3)	
b/Å	13.499(3)	
c/Å	13.640(3)	
α/°	107.58(3)	
β/°	99.50(3)	
γ/°	91.92(3)	
Volume/Å^3	2134.1(8)	
Z	2	
ρ_{calc}g/cm^3	1.180	
μ/mm^{-1}	0.598	
F(000)	788.0	
Crystal size/mm^3	0.1 × 0.08 × 0.05	
Radiation	(λ = 0.700)	
2Θ range for data collection/°	3.13 to 51.898	
Index ranges	-15 ≤ h ≤ 15, -16 ≤ k ≤ 16, -17 ≤ l ≤ 17	
Reflections collected	28811	
Independent reflections	8185 [R_{int} = 0.0415, R_{sigma} = 0.0368]	
Data/restraints/parameters	8185/0/360	
Goodness-of-fit on F^2	1.066	
Final R indexes [I>=2σ (I)]	R_1 = 0.0660, wR_2 = 0.2045	
Final R indexes [all data]	R_1 = 0.0777, wR_2 = 0.2140	
Largest diff. peak/hole / e Å^{-3}	1.48/-0.76	

Table S8. Bond lengths for [Zn(tpt)(L)$_2$]·2DMF (**3**).

Atom	Atom	Length/Å		Atom	Atom	Length/Å
Zn01	O002	2.2720		C00J	C011	1.4084
Zn01	O003	2.2235		C00J	C013	1.3718
Zn01	N005	2.1061		C00K	C00M	1.3842
Zn01	N006	2.1207		C00K	C00V	1.4069
Zn01	N008	2.1235		C00L	C00M	1.4696
Zn01	N009	2.0824		C00M	C00V^{2}	1.4221(16)
Zn01	C010	2.5744		C00N	C01A	1.4306
O002	C010	1.2645		C00O	C014	1.3554
O003	C010	1.2593		C00Q	C00S	1.4205
N004	C00E	1.3832		C00R	C00X	1.4008
N004	C00L	1.3648		C00R	C016^{3}	1.406(2)
N004	C018	1.4031		C00S	C00U	1.3574
N005	C00D	1.3115		C00T	C01F	1.3587
N005	C00I	1.3736		C00U	C014	1.4428
N006	C00N	1.3270		C00W	C00Y	1.3843
N006	C00T	1.3475		C00X	C016	1.3754
N007	C00D	1.3749		C00Y	C013	1.4182
N007	C00O	1.3760		C010	C016	1.4892
N007	C00Q	1.4038		C018	C01E	1.3489
N008	C00H	1.3684		C019	C01D	1.3211
N008	C00L	1.3159		C01A	C01I	1.3671
N009	C00W	1.3449		C01D	C01E	1.4635
N009	C011	1.3642		C01F	C01I	1.4138
C00C	C00D	1.4870		O1	C6	1.223(7)
C00C	C00G	1.3847		N5	C22	1.813(11)
C00C	C00P^{1}	1.3982(16)		N5	C21	1.368(9)
C00E	C00H	1.3729		N5	C20	1.263(10)
C00E	C019	1.4569		N7	C6	1.334(7)
C00G	C00P	1.3909		N7	C8	1.461(8)
C00H	C00N	1.4725		N7	C14	1.422(8)
C00I	C00Q	1.4014		O11	C20	1.254(11)
C00I	C011	1.4467				

^{1}-X,1-Y,-Z; ^{2}-X,2-Y,1-Z; ^{3}1-X,1-Y,1-Z

Table S9. Bond angles for [Zn(tpt)(L)$_2$]·2DMF (**3**).

Atom	Atom	Atom	Angle/˚		Atom	Atom	Atom	Angle/˚
O002	Zn01	C010	29.4		N005	C00I	C011	119.0
O003	Zn01	O002	58.7		C00Q	C00I	C011	132.8
O003	Zn01	C010	29.3		C013	C00J	C011	119.3
N005	Zn01	O002	143.0		C00M	C00K	C00V	121.4
N005	Zn01	O003	87.4		N004	C00L	C00M	124.9
N005	Zn01	N006	106.7		N008	C00L	N004	109.2
N005	Zn01	N008	119.5		N008	C00L	C00M	125.9
N005	Zn01	C010	115.8		C00K	C00M	C00L	121.5
N006	Zn01	O002	87.2		C00K	C00M	C00V^{2}	119.85(6)
N006	Zn01	O003	86.7		C00V^{2}	C00M	C00L	118.60(6)
N006	Zn01	N008	78.2		N006	C00N	C00H	115.3

N006	Zn01	C010	85.8		N006	C00N	C01A	121.5
N008	Zn01	O002	96.6		C01A	C00N	C00H	123.1
N008	Zn01	O003	151.9		C014	C00O	N007	118.5
N008	Zn01	C010	124.7		C00G	C00P	C00C[1]	119.00(6)
N009	Zn01	O002	88.0		N007	C00Q	C00S	117.9
N009	Zn01	O003	93.4		C00I	C00Q	N007	105.4
N009	Zn01	N005	79.0		C00I	C00Q	C00S	136.7
N009	Zn01	N006	174.4		C00X	C00R	C016[3]	119.51(7)
N009	Zn01	N008	99.4		C00U	C00S	C00Q	119.9
N009	Zn01	C010	91.6		N006	C00T	C01F	123.0
C010	O002	Zn01	88.7		C00S	C00U	C014	119.8
C010	O003	Zn01	91.0		C00K	C00V	C00M[2]	118.76(6)
C00E	N004	C018	121.8		N009	C00W	C00Y	124.2
C00L	N004	C00E	108.4		C016	C00X	C00R	120.9
C00L	N004	C018	129.4		C00W	C00Y	C013	116.9
C00D	N005	Zn01	136.1		O002	C010	Zn01	61.9
C00D	N005	C00I	109.0		O002	C010	C016	119.0
C00I	N005	Zn01	109.7		O003	C010	Zn01	59.7
C00N	N006	Zn01	114.0		O003	C010	O002	121.6
C00N	N006	C00T	119.2		O003	C010	C016	119.4
C00T	N006	Zn01	126.1		C016	C010	Zn01	176.4
C00D	N007	C00O	129.8		N009	C011	C00I	113.4
C00D	N007	C00Q	107.4		N009	C011	C00J	121.3
C00O	N007	C00Q	122.8		C00J	C011	C00I	125.3
C00H	N008	Zn01	110.4		C00J	C013	C00Y	120.0
C00L	N008	Zn01	138.6		C00O	C014	C00U	121.0
C00L	N008	C00H	108.1		C00R[3]	C016	C010	119.74(7)
C00W	N009	Zn01	126.7		C00X	C016	C00R[3]	119.50(7)
C00W	N009	C011	118.2		C00X	C016	C010	120.7
C011	N009	Zn01	114.9		C01E	C018	N004	120.5
C00G	C00C	C00D	120.9		C01D	C019	C00E	119.9
C00G	C00C	C00P[1]	120.39(6)		C01I	C01A	C00N	118.3
C00P[1]	C00C	C00D	118.70(6)		C019	C01D	C01E	121.7
N005	C00D	N007	110.1		C018	C01E	C01D	118.2
N005	C00D	C00C	125.7		C00T	C01F	C01I	118.6
N007	C00D	C00C	124.2		C01A	C01I	C01F	119.2
N004	C00E	C019	117.2		C21	N5	C22	108.6(6)
C00H	C00E	N004	105.1		C20	N5	C22	117.8(7)
C00H	C00E	C019	137.6		C20	N5	C21	133.2(8)
C00C	C00G	C00P	120.6		C6	N7	C8	119.6(5)
N008	C00H	C00E	109.2		C6	N7	C14	122.9(5)
N008	C00H	C00N	117.6		C14	N7	C8	117.4(5)
C00E	C00H	C00N	133.1		O1	C6	N7	125.5(6)
N005	C00I	C00Q	108.2		O11	C20	N5	114.6(9)

[1]-X,1-Y,-Z; [2]-X,2-Y,1-Z; [3]1-X,1-Y,1-Z
